# Genome Analysis of Two Pseudomonas syringae pv. *aptata* Strains with Different Virulence Capacity Isolated from Sugar Beet: Features of Successful Pathogenicity in the Phyllosphere Microbiome

**DOI:** 10.1128/spectrum.03598-22

**Published:** 2023-03-13

**Authors:** Tamara Ranković, Ivan Nikolić, Tanja Berić, Tatjana Popović, Jelena Lozo, Olja Medić, Slaviša Stanković

**Affiliations:** a University of Belgrade, Faculty of Biology, Center for Biological Control and Plant Growth Promotion, Belgrade, Serbia; b Institute for Plant Protection and Environment, Belgrade, Serbia; Pennsylvania State University

**Keywords:** draft genome, *Pseudomonas syringae* pv. *aptata*, bacterial plant pathogen, comparative genomics

## Abstract

Members of the Pseudomonas syringae species complex are heterogeneous bacteria that are the most abundant bacterial plant pathogens in the plant phyllosphere, with strong abilities to exist on and infect different plant hosts and survive in/outside agroecosystems. In this study, the draft genome sequences of two pathogenic P. syringae pv. *aptata* strains with different *in planta* virulence capacities isolated from the phyllosphere of infected sugar beet were analyzed to evaluate putative features of survival strategies and to determine the pathogenic potential of the strains. The draft genomes of P. syringae pv. *aptata* strains P16 and P21 are 5,974,057 bp and 6,353,752 bp in size, have GC contents of 59.03% and 58.77%, respectively, and contain 3,439 and 3,536 protein-coding sequences, respectively. For both average nucleotide identity and pangenome analysis, P16 and P21 largely clustered with other pv. *aptata* strains from the same isolation source. We found differences in the repertoire of effectors of the type III secretion system among all 102 selected strains, suggesting that the type III secretion system is a critical factor in the different virulent phenotypes of P. syringae pv. *aptata*. During genome analysis of the highly virulent strain P21, we discovered genes for T3SS effectors (AvrRpm1, HopAW1, and HopAU1) that were not previously found in genomes of P. syringae pv. *aptata*. We also identified coding sequences for pantothenate kinase, VapC endonuclease, phospholipase, and pectate lyase in both genomes, which may represent novel effectors of the type III secretion system.

**IMPORTANCE** Genome analysis has an enormous effect on understanding the life strategies of plant pathogens. Comparing similarities with pathogens involved in other epidemics could elucidate the pathogen life cycle when a new outbreak happens. This study represents the first in-depth genome analysis of Pseudomonas syringae pv. *aptata*, the causative agent of leaf spot disease of sugar beet. Despite the increasing number of disease reports in recent years worldwide, there is still a lack of information about the genomic features, epidemiology, and pathogenic life strategies of this particular pathogen. Our findings provide advances in disease etiology (especially T3SS effector repertoire) and elucidate the role of environmental adaptations required for prevalence in the pathobiome of the sugar beet. From the perspective of the very heterogeneous P. syringae species complex, this type of analysis has specific importance in reporting the characteristics of individual strains.

## INTRODUCTION

The phyllosphere represents a challenging habitat for many microbes, primarily due to constant fluctuations in environmental conditions, exposure to stress factors, and deficiency of nutrient sources (1). Like other members of the leaf microbiota (e.g., beneficial microbes), plant-pathogenic bacteria have to mitigate biotic and abiotic stressors by developing a set of mechanisms involved in motility, nutrient uptake, intermicrobial competition, and host plant manipulation (2). Phytopathogenic bacteria harbor a diverse weaponry of virulence factors, including the type III secretion system (T3SS) and its effectors, phytotoxins, phytohormones, ice nucleation activity (INA), plant cell wall-degrading enzymes, and exopolysaccharides (3).

Pseudomonas syringae inhabits diverse ecological niches and copes with different environmental conditions (4). Its prevalence in the leaf microbiota and the pathogenic potential of P. syringae are achieved chiefly via features such as (i) ubiquity, (ii) a broad host range, (iii) a diversity of virulence/competition strategies, and (iv) versatile genomic/metabolic features involved in host colonization (5). In the vocabulary used for pathogen diagnosis, P. syringae is characterized as a species complex comprised of several closely related Pseudomonas phytopathogenic species, which are similar at the genetic level (6). Based on pathogenic characteristics, more than 60 different pathovars have been described thus far (7). However, some experts emphasize the need to reconsider this terminology (8). Focusing on P. syringae pv. *aptata* (Ptt), the causative agent of leaf spot disease, frequent disease reports and the occurrence of new emergences recorded in recent years indicate that this bacterium is a potential candidate for future severe beet and chard field epidemics (9–12).

The P. syringae pv. *aptata* strains P16 and P21 were reported as causative agents of leaf spot disease on sugar beet in Serbia (13). In our previous studies, greenhouse experiments showed a difference in the two strains' pathogenic potential (virulence and host range) (8, 14). The P16 strain was characterized as a mildly low virulent strain with a narrow host range, while the P21 strain was depicted as one of a group of highly virulent strains with a very broad host range. The present study aimed to perform genomic profiling of these two plant pathogens by evaluating the genes that encode features involved in strategies necessary for survival and virulence capacity in the phyllosphere microbiome. This allows us to test the hypothesis that the different virulence associated gene repertoire of these two strains is responsible for the evident difference in pathogenicity detected *in planta*. Numerous research focused on the molecular biology and pathogenesis of plant-pathogenic bacteria, and the determinant factors in host-pathogen interactions have opened the door to a new era in bacterial disease management (15). Despite the increasing number of disease reports in recent years worldwide, there is still a lack of information in the literature about the genomic basis of pathogenic traits of P. syringae pv. *aptata*. To the best of our knowledge, this is the first in-depth analysis of P. syringae pv. *aptata* genomes for the purpose of elucidating overall features responsible for putative prevalence in the phyllosphere microbiome.

## RESULTS AND DISCUSSION

### General features of Pseudomonas syringae pv. *aptata* P16 and P21 draft genomes and their phylogenomic status within the Pseudomonas syringae complex.

To understand the differences between P. syringae pv. *aptata* isolates P16 and P21 and their phylogenetic status within the P. syringae species complex, we compared their genomes with the genomes of 100 strains from the NCBI database. The genomes selected for analysis were closely related strains from phylogenetic group (PG) 02, which consists of ubiquitous, heterogeneous, and highly pathogenic P. syringae strains. We also included in the overall comparison publicly available genomes of representative strains from PGs 01, 03, 04, 07, 09, 10, and 13 isolated from various environmental sources. According to NCBI data, a general comparison of genome sizes shows that the analyzed P. syringae strains have genome sizes in the range of 5.77 to 6.87 Mbp. Comparison of genome sizes within PG02 showed that the isolate P. syringae pv. *aptata* P21 is in the group of strains with the largest genome size (6.35 Mbp), similar to another strain of the same P. syringae pv. *aptata* pathovar, namely, DSM50252 (6.36 Mbp). Strains P16 and P21 had far more tRNA (56) and rRNA genes (9) than other strains (tRNA 29 to 44; rRNA 3 to 4) from the same pathovar (Table 1). The general features and assembly statistics of the draft genomes of P. syringae pv. *aptata* strains P16 and P21 and other 100 P. syringae genomes included in the phylogenomic comparison are listed in Table 1. To validate annotation results and assess annotated gene homology, we searched for the blast hits between two genomes from our strains and reference strain P. syringae pv. *syringae* B728a, as a widely used model system and member of PG02. We revealed 5402 CDSs hits between P21 and B728a, and 4,181 CDSs hits between P16 and B728a with a percentage identity above 80%. The CDS were classified into different groups (COG categories) based on their roles in the cell (Table 2). Among CDSs with recognized function, 597 CDSs for the P16 strain and 653 CDSs for the P21 strain did not have any COG category.

**TABLE 1:** General information of genomes used for comparisons between P. syringae strains and phylogenetic analysis

Name	Strain	Phylogenetic group	Host (isolation source)	Geographic location	GenBank assembly accession	Genome size (bp)	GC %	Protein coding sequences	tRNK coding genes	rRNK
Pseudomonas syringae *pv. aptata*	P16	PG02	*Beta vulgaris*	Serbia	GCA_018642105.1	5,974,057	59.0	5,360	56	9
Pseudomonas syringae *pv. aptata*	P21	PG02	*Beta vulgaris*	Serbia	GCA_018530765.1	6,353,752	58.8	5,727	56	9
Pseudomonas syringae *pv. aptata*	G733	PG02	Oryza sativa	-	GCA_003699535.1	5,802,565	59.3	5,006	44	3
Pseudomonas syringae *pv. aptata*	ICMP11935	PG02	*Beta vulgaris*	France	GCA_003702445.1	5,984,734	58.7	4,700	29	3
Pseudomonas syringae *pv. aptata*	DSM50252	PG02	*Beta vulgaris*	-	GCA_000145905.1	6,364,678	59.1	6,364	36	4
Pseudomonas syringae *pv. aptata*	ICMP459	PG02	*Beta vulgaris*	USA	GCA_001401335.1	5,977,364	58.8	5,005	44	3
Pseudomonas syringae *pv. aptata*	ICMP4388	PG02	*Beta vulgaris*	USA	GCA_003700795.1	5,859,953	58.7	4,533	30	3
Pseudomonas syringae *pv. aceris*	M302273	PG02	*Acer sp.*	USA	GCA_000145925.1	6,250,569	59.0	5,600	40	3
Pseudomonas syringae *pv. aceris*	A10853	PG02	*Acer sp.*	USA	GCA_001270465.1	6,285,198	59.0	5,341	42	3
Pseudomonas syringae *pv. aceris*	ICMP9850	PG02	*Acer buergerianum*	Japan	GCA_003700545.1	5,873,352	59.0	5,002	40	4
Pseudomonas syringae *pv. aceris*	ICMP9852	PG02	*Acer buergerianum*	Japan	GCA_003700575.1	5,777,533	59.0	4,954	52	4
Pseudomonas syringae *pv. aceris*	ICMP9851	PG02	*Acer buergerianum*	Japan	GCA_003701675.1	5,809,409	59.0	4,905	37	3
Pseudomonas syringae *pv. aceris*	ICMP2802	PG02	*Acer sp.*	-	GCA_001400655.1	6,300,961	59.0	5,486	53	4
Pseudomonas syringae *pv. Atrofaciens*	LMG5059	PG02	Triticum aestivum	New Zealand	GCA_003047185.1	6,080,544	58.9	5,060	67	16
Pseudomonas syringae *pv. Atrofaciens*	DSM5025	PG02	Triticum aestivum	USA	GCA_003699335.1	5,848,565	59.0	5,008	46	3
Pseudomonas syringae *pv. Atrofaciens*	ICMP4394	PG02	Triticum aestivum	New Zealand	GCA_001400125.1	6,012,246	58.5	5,129	57	4
Pseudomonas syringae *pv. Atrofaciens*	ICMP5011	PG02	Triticum aestivum	Zimbabwe	GCA_003699595.1	5,890,334	59.0	5,107	57	4
Pseudomonas syringae *pv. Atrofaciens*	ICMP1852	PG02	Triticum aestivum	New Zealand	GCA_003700175.1	5,851,851	59.0	5,384	44	3
Pseudomonas syringae *pv. Atrofaciens*	DSM50255	PG02	Triticum aestivum	USA	GCA_000498595.1	5,777,269	59.0	5,204	31	3
Pseudomonas syringae *pv. dysoxyli*	CFBP2356	PG02	*Dysoxylum spectabile*	New Zealand	GCA_009898305.1	5,944,509	59.0	5,023	55	7
Pseudomonas syringae *pv. japonica*	M301072	PG02	-*	USA	GCA_000145785.1	6,380,619	59.0	8,796	48	6
Pseudomonas syringae *pv. lapsa*	ATCC10859	PG02	Triticum aestivum	-	GCA_001482725.1	5,918,899	59.1	4,973	63	16
Pseudomonas syringae *pv. lapsa*	ICMP3946	PG02	-	-	GCA_003698895.1	5,856,122	59.0	5,010	42	4
Pseudomonas syringae *pv. lapsa*	ICMP3947	PG02	*Zea sp.*	-	GCA_001400495.1	5,850,429	59.0	5,003	42	2
Pseudomonas syringae *pv. lapsa*	ICMP8813	PG02	-	-	GCA_003699285.1	5,838,357	59.0	5,022	40	2
Pseudomonas syringae *pv. papulans*	CFBP1754	PG02	*Malus sylvestris*	Canada	GCA_001535905.1	6,180,564	58.5	5,467	60	9
Pseudomonas syringae *pv. papulans*	ICMP4040	PG02	*Malus domestica*	USA	GCA_003699835.1	6,100,320	58.5	5,370	48	3
Pseudomonas syringae *pv. papulans*	ICMP4048	PG02	*Malus domestica*	Canada	GCA_001401005.1	6,097,472	58.5	5,341	45	3
Pseudomonas syringae *pv. papulans*	ICMP4050	PG02	*Malus domestica*	Canada	GCA_003702795.1	6,128,836	58.5	5,381	42	3
Pseudomonas syringae *pv. papulans*	ICMP4986	PG02	*Malus domestica*	USA	GCA_003699185.1	6,351,666	58.5	5,751	44	3
Pseudomonas syringae *pv. pisi*	PP1	PG02	Pisum sativum	Japan	GCA_000452445.3	6,039,562	58.8	5,136	64	16
Pseudomonas syringae *pv. pisi*	202	PG02	Pisum sativum	USA	GCA_003205965.1	6,505,845	58.0	5,671	62	7
Pseudomonas syringae *pv. pisi*	203	PG02	Pisum sativum	New Zealand	GCA_003205895.1	6,576,361	57.5	5,762	62	6
Pseudomonas syringae *pv. pisi*	895A	PG02	Pisum sativum	-	GCA_003699325.1	5,997,727	58.5	5,180	47	4
Pseudomonas syringae *pv. pisi*	H6E5	PG02	Pisum sativum	-	GCA_003699005.1	6,150,060	58.5	5,347	47	4
Pseudomonas syringae *pv. pisi*	H5E3	PG02	Pisum sativum	-	GCA_003699035.1	5,951,392	58.5	5,163	45	4
Pseudomonas syringae *pv. pisi*	ICMP2788	PG02	Pisum sativum	USA	GCA_003700675.1	6,282,175	58.0	5,443	42	4
Pseudomonas syringae *pv. pisi*	R6a	PG02	Pisum sativum	-	GCA_003698965.1	6,185,107	58.5	5,399	41	3
Pseudomonas syringae *pv. pisi*	ICMP4433	PG02	Pisum sativum	Canada	GCA_003703105.1	6,175,014	58.0	5,351	35	3
Pseudomonas syringae *pv. pisi*	1704B	PG02	Pisum sativum	-	GCA_000145805.1	6,520,586	-	9,160	48	3
Pseudomonas syringae *pv. solidagae*	ICMP16927	PG02	*Solidago canadensis*	Japan	GCA_003701885.1	5,938,726	59.0	5,028	38	3
Pseudomonas syringae *pv. solidagae*	ICMP16925	PG02	*Solidago altissima*	Japan	GCA_001401055.1	5,980,445	59.0	5,087	33	3
Pseudomonas syringae *pv. solidagae*	ICMP16926	PG02	*Solidago canadensis*	Japan	GCA_003701925.1	5,924,161	59.0	5,052	32	3
Pseudomonas syringae *pv. syringae*	CFBP4215	PG02	Prunus avium	France	GCA_900235825.1	6,035,297	59.3	4,984	63	16
Pseudomonas syringae *pv. syringae*	B728a	PG02	Phaseolus vulgaris	USA	GCA_000012245.1	6,093,698	59.2	5,072	63	16
Pseudomonas syringae *pv. syringae*	9097	PG02	*Prunus sp.*	United Kingdom	GCA_002905815.2	5,929,959	59.3	4,886	63	15
Pseudomonas syringae *pv. syringae*	HS191	PG02	*Proso millet*	Australia	GCA_000988395.1	6,002,759	58.9	5,065	65	16
Pseudomonas syringae *pv. syringae*	CFBP2118	PG02	*Prunus cerasus*	France	GCA_900235865.1	6,031,285	59.3	4,983	63	16
Pseudomonas syringae *pv. syringae*	B301D	PG02	Pyrus communis	United Kingdom	GCA_000988485.1	6,094,819	59.2	5,095	63	16
Pseudomonas syringae *pv. syringae*	UMAF0158	PG02	Mangifera indica	Spain	GCA_001281365.1	5,850,990	59.3	4,973	63	16
Pseudomonas syringae *pv. syringae*	SM	PG02	Triticum aestivum	USA	GCA_000412165.1	6,094,398	59.0	5,172	64	16
Pseudomonas syringae *pv. syringae*	B64	PG02	Triticum aestivum	-	GCA_000331385.1	5,873,298	59.0	4,938	61	13
Pseudomonas syringae *pv. syringae*	ICMP3023	PG02	*Syringa vulgaris*	United Kingdom	GCA_001401075.1	6,073,080	59.0	5,127	56	4
Pseudomonas syringae *pv. syringae*	9656	PG02	Prunus avium	United Kingdom	GCA_002905975.1	5,980,728	59.0	5,015	55	9
Pseudomonas syringae *pv. syringae*	100	PG02	*Phaseolus lunatus*	Kenya	GCA_002917175.1	5,872,916	59.0	4,999	57	9
Pseudomonas syringae *pv. syringae*	3023	PG02	*Syringa vulgaris*	United Kingdom	GCA_002917245.1	6,203,212	59.0	5,250	60	9
Pseudomonas syringae *pv. syringae*	41A	PG02	*Prunus armeniaca*	France	GCA_000935775.1	5,983,849	59.0	5,027	52	5
Pseudomonas syringae *pv. syringae*	UMAF0291	PG02	Mangifera indica	Spain	GCA_014493645.1	5,857,022	59.0	4,908	57	9
Pseudomonas syringae *pv. syringae*	UMAF3028	PG02	Mangifera indica	Spain	GCA_014493635.1	5,856,306	59.0	4,908	60	8
Pseudomonas syringae *pv. syringae*	UMAF0081	PG02	Mangifera indica	Spain	GCA_023232565.1	5,874,868	59.0	4,936	58	11
Pseudomonas syringae *pv. syringae*	5264	PG02	Prunus avium	United Kingdom	GCA_002916225.1	6,029,896	59.0	5,150	58	10
Pseudomonas syringae *pv. syringae*	LMG 5084	PG02	Pyrus communis	United Kingdom	GCA_013416855.1	6,144,879	59.0	5,122	55	13
Pseudomonas syringae *pv. syringae*	5275	PG02	Prunus avium	United Kingdom	GCA_002916275.1	5,994,461	59.0	5,051	55	5
Pseudomonas syringae *pv. syringae*	8094A	PG02	Prunus avium	United Kingdom	GCA_002916355.1	5,942,438	59.0	4,996	57	9
Pseudomonas syringae *pv. syringae*	2676C	PG02	Phaseolus vulgaris	Lesotho	GCA_002917155.1	6,158,476	59.0	5,195	55	8
Pseudomonas syringae *pv. syringae*	2340	PG02	*Pyrus sp.*	Hungary	GCA_001535725.1	6,181,159	59.0	5,213	58	13
Pseudomonas syringae *pv. syringae*	9654	PG02	*Prunus domestica*	United Kingdom	GCA_002906015.1	5,941,610	59,0	4,984	55	8
Pseudomonas syringae *pv. syringae*	2682C	PG02	Phaseolus vulgaris	Lesotho	GCA_002917215.1	6,259,099	59.0	5,306	55	8
Pseudomonas syringae *pv. syringae*	2675C	PG02	*Abelmoschus esculentus*	Kenya	GCA_002917195.1	5,994,384	59.0	4,999	57	12
Pseudomonas syringae *pv. syringae*	NZIPFR-PS7	PG02	Prunus avium	New Zealand	GCA_002736945.1	6,143,879	59.0	5,187	57	7
Pseudomonas syringae *pv. syringae*	NZIPFR-IHC1	PG02	*Prunus sp.*	New Zealand	GCA_002736965.1	6,090,296	59.0	5,111	65	13
Pseudomonas syringae *pv. syringae*	HRI-W 7872	PG02	*Prunus domestica*	United Kingdom	GCA_001535875.1	5,899,795	59.0	4,950	55	11
Pseudomonas syringae *pv. syringae*	MB03	PG02	*Populus lasiocarpa*	China	GCA_001623415.1	5,777,225	59.0	4,915	53	4
Pseudomonas syringae *pv. syringae*	A1M244	PG02	Prunus avium	Chile	GCA_022510265.1	5,979,725	59.0	4,999	53	4
Pseudomonas syringae *pv. syringae*	2339	PG02	Prunus avium	Hungary	GCA_001535855.1	6,122,357	59.0	5,098	56	12
Pseudomonas syringae *pv. syringae*	1845	PG02	*Helianthus annuus*	Russia	GCA_001675415.1	5,768,876	59.0	4,773	58	5
Pseudomonas syringae *pv. syringae*	2507	PG02	Triticum aestivum	Russia	GCA_001675375.1	5,944,881	59.0	4,929	54	4
Pseudomonas syringae *pv. syringae*	Alf3	PG02	*Alfalfa*	USA	GCA_000738515.1	5,806,984	59.0	4,912	46	3
Pseudomonas syringae *pv. syringae*	A2	PG02	*Pyrus calleryana*	-	GCA_001293665.1	5,901,107	59.0	5,019	39	3
Pseudomonas syringae *pv. syringae*	B48	PG02	*Prunus persica*	USA	GCA_001293705.1	5,975,993	59.0	5,083	42	3
Pseudomonas syringae *pv. syringae*	ICMP4917	PG02	*Citrus limon*	France	GCA_003702385.1	5,928,691	59.0	5,097	41	4
Pseudomonas syringae *pv. syringae*	ICMP3688	PG02	Prunus dulcis	New Zealand	GCA_003700375.1	5,805,147	59.0	4,941	42	4
Pseudomonas syringae *pv. syringae*	127	PG02	Prunus avium	South Africa	GCA_002611605.1	6,083,167	59.0	5,444	40	2
Pseudomonas syringae *pv. syringae*	126	PG02	Prunus avium	South Africa	GCA_002611615.1	5,953,004	59.0	5,006	48	4
Pseudomonas syringae *pv. syringae*	545	PG02	Phaseolus vulgaris	Canada	GCA_003412685.1	5,702,919	59.0	4,961	28	3
Pseudomonas syringae *pv. syringae*	PD2774	PG02	*Actinidia sp.*	USA	GCA_001466875.1	6,357,389	59.0	5,365	52	5
Pseudomonas syringae *pv. syringae*	B301D-R	PG02	Pyrus communis	United Kingdom	GCA_000585725.1	6,036,561	59.0	5,071	54	9
Pseudomonas syringae *pv. syringae*	1212	PG02	Pisum sativum	United Kingdom	GCA_000452465.2	6,163,906	59.0	5,284	53	4
Pseudomonas syringae *pv. syringae*	9293	PG02	*Prunus domestica*	United Kingdom	GCA_002905935.1	6,135,031	59.0	5,336	55	8
Pseudomonas syringae *pv. syringae*	9630	PG02	*Prunus domestica*	United Kingdom	GCA_002905995.1	5,940,819	59.0	5,115	57	11
Pseudomonas syringae *pv. syringae*	9644	PG02	Prunus avium	United Kingdom	GCA_002905835.1	6,173,193	59.0	5,245	58	11
Pseudomonas syringae *pv. syringae*	9659	PG02	Prunus avium	United Kingdom	GCA_002905855.1	5,943,090	59.0	5,090	54	8
Pseudomonas syringae *pv. syringae*	NZIPFR-VIR1	PG02	Prunus avium	New Zealand	GCA_002736905.1	6,875,296	59.0	3,705	61	7
Pseudomonas syringae *pv. syringae*	WSPS007	PG02	*Malus domestica*	South Korea	GCA_004005515.1	5,498,736	59.5	5,134	-	-
Pseudomonas syringae	USA011	PG02	*Stream water*	USA	GCA_000452525.4	6,112,257	59.2	5,051	62	16
*Pseudomoans syringae pv. Tomato*	DC3000	PG01	*Solanum lycopersicum*	United Kingdom	GCA_000007805.1	6,538,260	58.3	5,576	63	16
Pseudomonas syringae *pv. Morsprunorum*	CFBP 2116	PG03	*Prunus cerasus*	France	GCA_900289125.1	6,262,486	58.0	5,385	65	16
Pseudomonas tremae	CC1513	PG04	*Pritzelago alpina*	France	GCA_000452765.1	5,725,032	57.5	5,094	39	3
Pseudomonas viridiflava	CC1582	PG07	Epilithon	France	GCA_000452505.1	5,985,404	59.0	5,299	30	2
Pseudomonas syringae	CC1417	PG09	Epilithon	USA	GCA_000452825.2	5,648,464	590	4,988	41	3
Pseudomonas syringae	CC1557	PG10	Snow	France	GCA_000452705.3	5,811,653	58.5	4,919	62	16
Pseudomonas syringae	UB246	PG13	River water	France	GCA_000452865.1	6,176,522	57.0	5,509	28	2

**TABLE 2:** Number of annotated gene functions according to COG functional classification

Functional categories of genes	No. of genes	
	P16	P21
A - RNA processing and modification	1	1
B - Chromatin structure and dynamics	0	0
C - Energy production and conversion	141	142
D - Cell cycle control, cell division, chromosome partitioning	32	32
E - Amino acid transport and metabolism	219	212
F - Nucleotide transport and metabolism	61	57
G - Carbohydrate transport and metabolism	134	131
H - Coenzyme transport and metabolism	120	117
I - Lipid transport and metabolism	105	101
J - Translation, ribosomal structure, and biogenesis	198	201
K - Transcription	126	123
L - Replication, recombination, and repair	99	99
M - Cell wall/membrane/envelope biogenesis	143	148
N - Cell motility	35	36
O - Posttranslational modification, protein turnover, chaperones	105	103
P - Inorganic ion transport and metabolism	144	140
Q - Secondary metabolites biosynthesis, transport, and catabolism	36	36
R - General function prediction only	69	65
S - Function unknown	25	28
T - Signal transduction mechanisms	117	119
U - Intracellular trafficking, secretion and vesicular transport	35	36
V - Defense mechanisms	39	40
W - Extracellular structures	0	0
X - Mobilome: prophages, transposons	3	6
Y - Nuclear structure	0	0
Z - Cytoskeleton	1	1
Not in COGs	597	653

Phylogenetic studies based on multilocus sequence analysis (MLSA) of several housekeeping genes have made a significant contribution by determining the status of particular pathovars and strains and by establishing the existence of 13 phylogenetic groups within the P. syringae complex (16–18). Previous phylogenetic analysis revealed that P. syringae pv. *aptata* belongs to the 02 phylogenetic group (and subgroup 02b), consisting of ubiquitous strains with strong epiphytic phases (16). Phylogenomic studies within the P. syringae complex provided a thorough analysis of the P. syringae complex using publicly available genome data (7, 19, 20). Six phylogenomic branches were distinguished based on 139 draft and complete genomes of P. syringae species complex, grouping the P. syringae pv. *aptata* DSM 50252 strain in the phylogenomic branch I, mainly consisting of members belonging to the PG02 phylogenetic group (7).

### Average nucleotide identity analysis.

**FIG 1:**
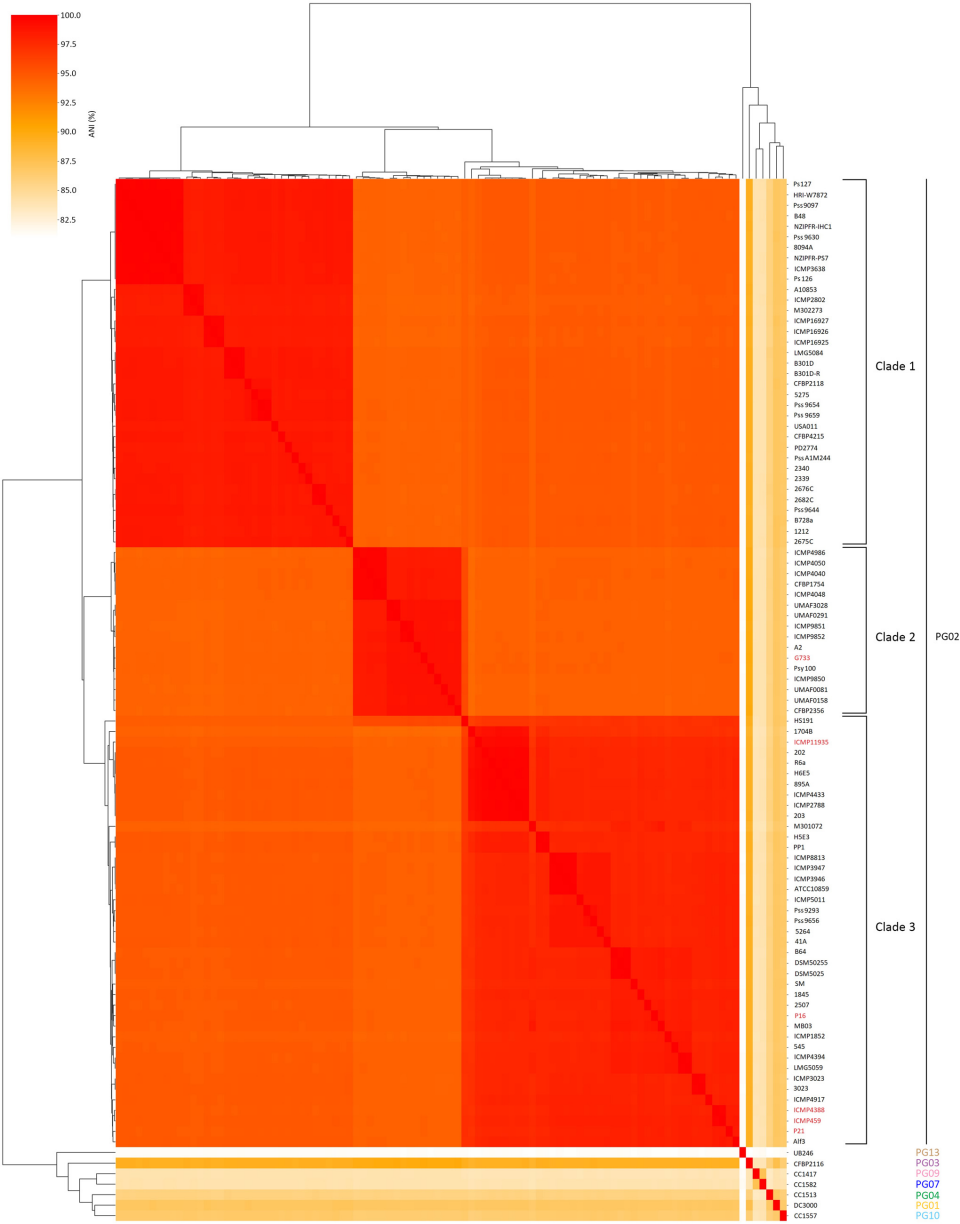
Average nucleotide identity (ANI) dendrogram of 99 strains within the P. syringae complex. The genome comparisons were made using FastANI, with a 95% ANI cutoff value.

To estimate the average nucleotide identity between P. syringae pv. *aptata* P16 and P21 and other members of the P. syringae complex, pairwise alignment of genome sequences and similarity analysis were performed. Regarding the threshold, three different clades with a similarity level of at least 95% were observed within the strains of PG02 (Fig. 1). Isolates P16 and P21 were grouped in clade 3 with other P. syringae pv. *aptata* strains isolated from sugar beet and strains designated as members of P. syringae pathovars: *syringae*, *pisi*, *lapsa*, *japonica* and *atrofaciens* (average nucleotide identity [ANI] values 98.3% to 99.9%). Members of clade 2 represent P. syringae strains designated as pathovars *syringae*, *papulans*, *aceris*, and *dysoxily* (98.3% to 99.8%). P. syringae pv. *aptata* strain G733, isolated from rice, is also a member of this clade 2 with ANI values of 98.5% to 99.0% with all other members of clade 2. However, the ANI similarities with all P. syringae pv. *aptata* strains are lower, 94.4% to 94.6%. Clade 1 consists only of strains from P. syringae pathovars *syringae* and *solidagae* (ANI values 98.2% to 99.9%). Strains from other PGs differ significantly among themselves and from members of PG02 (ANI values 81.1% to 90.1%), justifying classification to separate phylogenetic groups. For example, strain UB246 (PG13) isolated from river water has lower ANI similarity (ANI values 81.1% to 81.5%) than all other 101 strains tested. The ANI represents robust methods for evaluating phylogenomic status and divergence among closely related strains (21, 22). Still, we observed certain inconsistencies in clear boundaries between previously obtained phylogenetic/phylogenomic groups and subgroups and proposed distinctive pathovars. Although taxonomic and phylogenomic analyzes of the P. syringae species complex were the focus of numerous studies, the divergence of approaches that use deposited genomic data could lead, to a certain extent, to noncompliance with previous similar studies. Many isolates from the databases have been named without full description, or their identifications haven't been updated and revised upon the novel and modern techniques development (7). This suggests the need for comprehensive harmonization of P. syringae genomic databases to provide solid genomic-based identification and comparison. Furthermore, our results show the great necessity to reconsider the genome-driven revision of P. syringae pathovars classification. Future development of more powerful genomic methods and analyzes will allow us to elucidate the epidemiology and disease etiology of P. syringae.

### Pangenome analysis.

**FIG 2:**
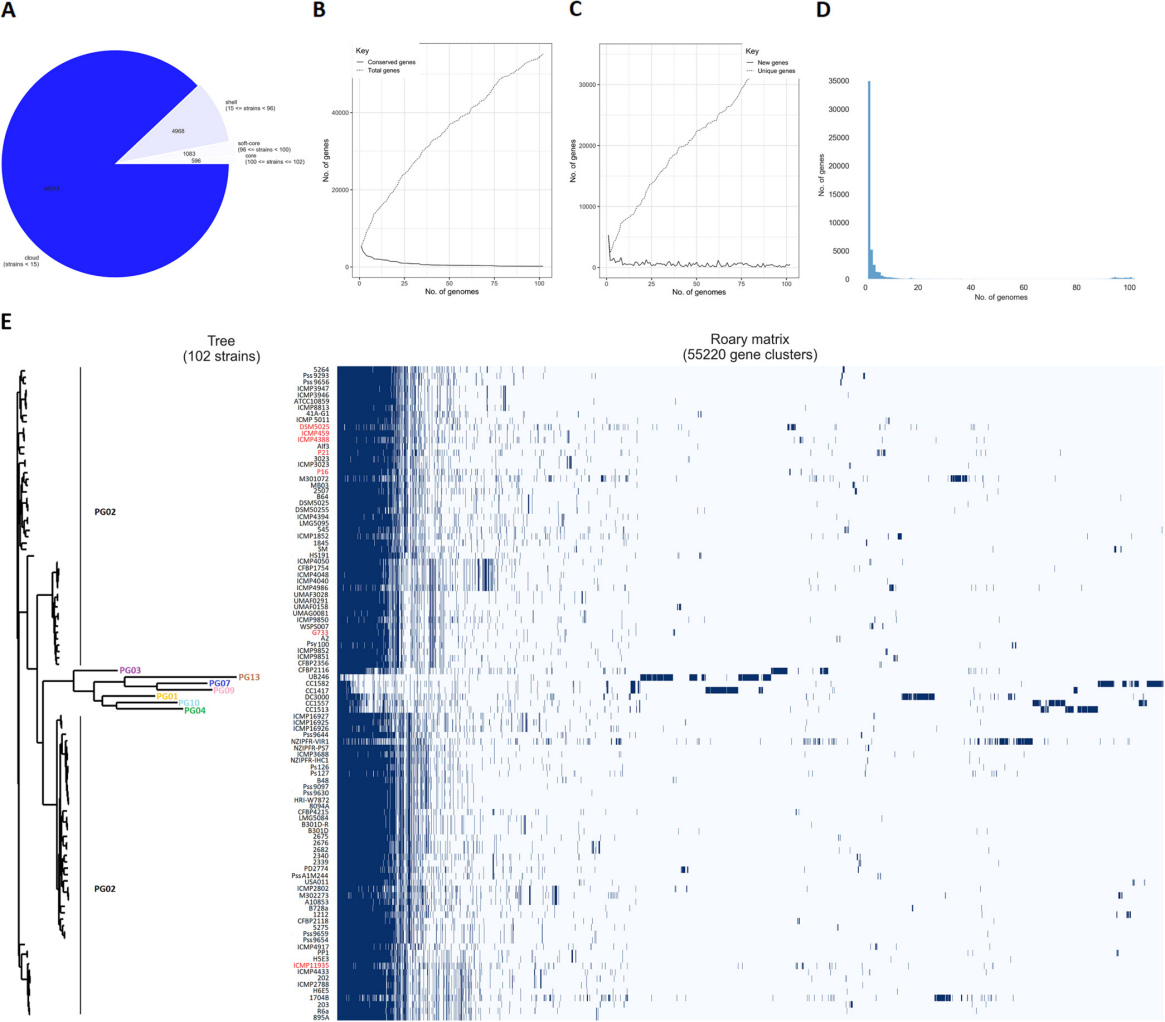
Pangenome analysis of 102 strains from P. syringae complex. (A) A pie chart represents the number of genes belonging to core, soft-core, shell and cloud genomes. (B) The size of the core genome (continuous line) and the pangenome (dashed line) in relation to the number of genomes compared. (C) The number of new genes (continuous line) and unique genes (dashed line) in relation to the number of genomes compared. (D) The frequency of genes versus the number of P. syringae genomes. (E) Gene presence/absence matrix shows the distribution of genes found in each genome. Dark blue blocks represent the presence of a gene, while white blocks indicate its absence. The approximately maximum likelihood phylogenetic tree was built based on core genome alignment. The strains from this study are indicated in red.

The pangenome of two isolates from this study and 100 P. syringae strains from the NCBI database contained 55,220 genes, of which 596 core, 1,083 soft-core, 4,968 shell, and 48,573 cloud genes (Fig. 2A). Core genes include highly conserved genes with phylogenetic information, while accessory genes (shell and cloud) are a flexible part of the genome (7). The obtained core genome comprised only 3.04% of the total pangenome, while a high percentage of accessory genes (97%) was observed. With more sampled genomes of P. syringae, the number of shell and cloud genes is expected to increase (6). The rarefaction curve of core/total genes showed that the number of core genes rapidly declined until 30 genomes were added, and subsequently curve remains fairly constant, while the number of total genes in the pan-genome continues to increase almost linearly (Fig. 2B). Additionally, the number of unique genes constantly increased with more sampled genomes (Fig. 2C). The frequency of genes within a whole-genome set showed that 35,000 genes were strain-specific (Fig. 2D). P. syringae strains have formed four separate clusters on the phylogenetic tree, and the distribution of accessory genes varied among the strains (Fig. 2E). According to core genome sequence analysis, the P16 and P21 were closely related and belonged to the same cluster with four other P. syringae pv. *aptata* strains, all originated from sugar beet. In contrast, P. syringae pv. *aptata* G733 was clustered in a separate clade as other P. syringae pv. *aptata* strains, along with P. syringae strains similar to ANI-clade 2 (Fig. 2E), suggesting that strain G733 was misidentified as P. syringae pv. *aptata*. The observed extensive accessory genome revealed a high degree of variability among different P. syringae strains (Fig. 2E), which could be associated with their ability to survive in diverse ecological niches and the extensive horizontal gene transfer throughout the P. syringae complex (6). On the other hand, core genome components are less likely to undergo horizontal gene transfer, which makes them more reliable for tracking the evolutionary history (23). A high percentage of the flexible genome (93.71%) was also previously observed in the pangenome analysis of 127 P. syringae genomes (7).

### Pathogenic features of P. syringae pv. *aptata* P16 and P21—plant colonization traits.

**FIG 3:**
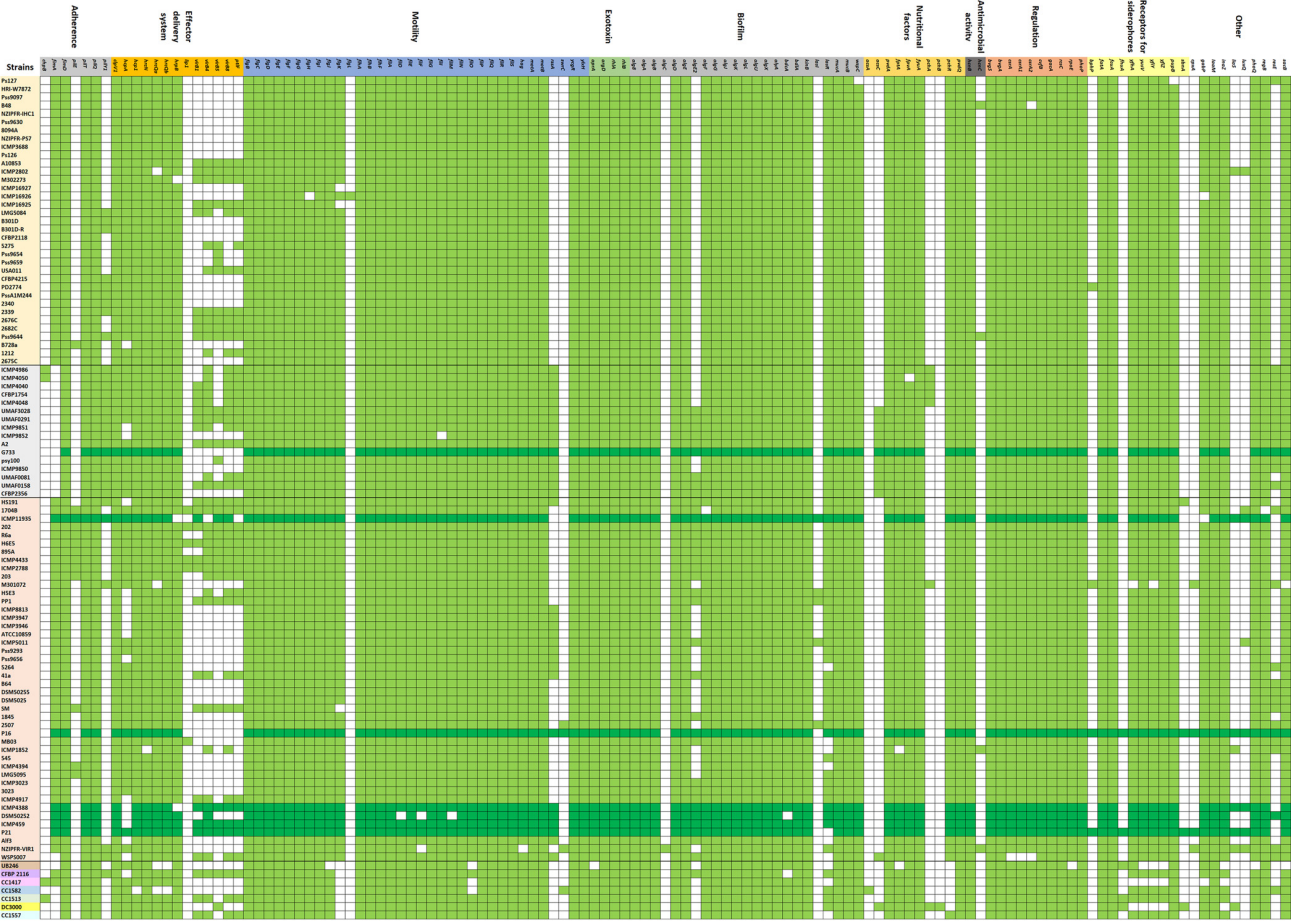
Representation of presence/absence of genes involved in colonization and virulence traits in P. syringae complex. P. syringae strains were ordered as in the ANI analysis. Strains marked in yellow, gray, and orange colors belong to ANI clades 1, 2, and 3 of PG02, respectively. Other strains are representatives of PGs 13, 03, 09, 07, 04, 01, and 10. Green blocks (dark green for pv. *aptata* strains) represent the presence of each gene and white blocks its absence.

Colonization of the phyllosphere and population fitness of the foliar plant pathogens represents critical factors for the successful development of epiphytic and endophytic life stages. To colonize (survive or infect) host plants, P. syringae has evolved numerous adaptations and features. Flagellar-dependent or -independent motility plays a crucial role in coping with variable environmental conditions and diverse interactions with plants (24). Swarming represents flagellar-mediated motility, which is required for movement on solid surfaces. It is an important trait for successfully colonizing the habitat by spreading a biofilm in different pathogenic varieties of the P. syringae complex (25). Based on the report of the presence/absence of genes from the pangenome analysis, we found that all P. syringae strains tested, including the P. syringae pv. *aptata* strains, contained genes putatively encoding flagella maintenance (the *flh*, *flg*, and *fli* genes) and the *che* chemotaxis genes (Fig. 3). In addition, P16 and P21 strains possess genes involved in swarming motility and twitching (flagellar-independent) motility. In particular, P16 contains genes that encode the swarming motility protein and the swarming regulation sensor protein (*swrC* and *rssA*), while only the *rssA* gene for swarming regulation was detected in P21 and other Ptt strains tested (Fig. 3).

Interestingly, swarming motility can be regulated by different environmental factors. Swarming in P. syringae pv. *syringae* B728a is light-mediated via histidine kinase (LOV-HK) as a positive regulator in response to blue light and a bacteriophytochrome (Bphp1) as a negative regulator in response to red/far-red light (26). Also, a study of thermal-mediated regulation of swarming motility in PssB728a showed that swarming is suppressed at 28 to 30°C, thereby affecting optimal growth temperature (27). All tested Ptt strains have a myriad of histidine kinases, while P16 and P21 also possess Bphp1, suggesting the possibility of light regulation of swarming in P. syringae pv. *aptata*.

Twitching motility represents type 4 pili (T4P)-mediated (flagellar-independent) movement, primarily used to move across wet and solid leaf surfaces (28). The assembly and functionality of T4P require around 40 genes that encode the major structural proteins involved in elongation and retraction (29). In the P. syringae complex, the best-studied organism with active twitching motility is P. syringae pv. *tabaci* 6605 (30, 31), although many other pathovars have T4P. The draft genomes of all analyzed P. syringae strains, including P. syringae pv. *aptata*, revealed a set of *pil* genes involved in the assembly, extension, attachment, and retraction of T4P and the *pilT* gene coding for the twitching motility protein (Fig. 3). That strongly suggests that P. syringae pv. *aptata* could use twitching motility to succeed in habitat colonization. Once reaching a comfortable area, bacterial pili allow adhesion and the initiation of biofilm formation, while the *bdlA* gene plays a key role in biofilm dispersion in P. syringae pv. *aptata*. It should be noted that swarming motility and T4P components are co-related with biofilm formation.

As a colonization factor in plant-pathogenic bacteria, the ability to form a biofilm requires the production of different biofilm matrix exopolysaccharides (EPSs) (32). In the draft genomes of neither of the strains from this study did we find putative genes for EPSs, except in the case of several P. syringae strains, including P16, which possesses only genes (*acsAC*) for cellulose synthases (Fig. 3). This putative trait of the P16 strain could be linked with low virulence activity in previous greenhouse testing, because it has been shown that cellulose reduces virulence in the P. syringae pv. *syringae* UMAF0158 strain (33). Presumably, cellulose could play a role in slowing down the transition from the epiphytic to the endophytic phase for P. syringae strains (34).

### Pathogenic features of P. syringae pv. *aptata* P16 and P21—plant virulence traits.

**FIG 4:**
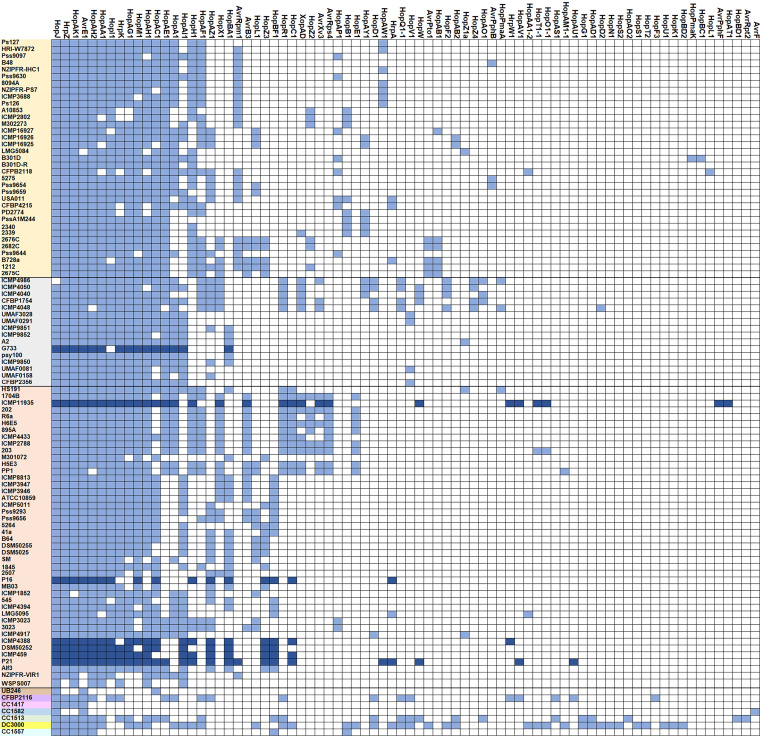
T3SS effectors repertoire detection by Effectidor analyzes. P. syringae strains were ordered as in the ANI analysis. Strains marked in yellow, gray, and orange colors belong to ANI clade 1, 2, and 3 of PG02, respectively. Other strains are representatives of PGs 13, 03, 09, 07, 04, 01, and 10. Certain effectors presence is indicated by blue color (dark blue for pv. *aptata* strains) and white blocks indicate its absence.

Plant pathogens use various mechanisms to regulate and express a large facet of genes required for virulence, manipulating the plant immune system and producing secondary metabolites. Virulence itself requires the engagement of genes encoding proteins that have a direct impact on host cell death (through T3SS and toxin production, ice nucleation activity) or mechanisms that create a virulence-like environment (uptake of γ-aminobutyric acid [GABA], alginate production). We performed a predictive machine-learning-based analysis to identify T3SS effectors in 102 P. syringae strains, including P16 and P21 strains. We were able to reveal the repertoire of 78 effectors in all strains, with 15 to 25 effectors detected in most strains (Fig. 4). The effectors in 90% of the analyzed strains studied were HopJ, HrpZ, HopAK1, AvrE1, HopAH2, HopAA1, HopI1, HrpK, HopAG1, and HopM1. In general, the P. syringae effectorome contains more than 70 effector families, of which only three, AvrE, HopM, and HopAA, are encoded in the conserved region of the canonical pathogenicity island and appear to be present in multiple strains of the P. syringae complex (35). The highest number of T3SS effectors (39 effectors) in our analysis was detected in the genome of the DC3000 strain. Only five other strains, including two Ptt strains (ICMP11935 and P21), had more than 25 effectors. On the other hand, strains isolated from environmental sources (nonplant associated) have a small effector repertoire (e.g., only three known effectors were detected in strains UB246 and CC1582).

Pseudomonas syringae pv. *aptata* was previously established as a pathovar with a small repertoire of T3S effector genes (36). In the comparative genomic study of 19 P. syringae pathovars, P. syringae pv. *aptata* strain DSM50252 contained only 12 T3S effector biosynthetic genes (36). Our analysis revealed 16 previously known effectors in DSM50252, whereas 29 effectors were detected in ICMP11935 (Fig. 4). Of all the strains compared, the effectors AvrPphF and HopAT1 were detected only in ICMP11935. Overall, analysis of all six P. syringae pv. *aptata* strains isolated from sugar beet revealed the presence of 32 different T3SS effectors, indicating a significantly larger repertoire of effectors than previously known. The genomes of Ptt strains, including P16 and P21 strains, contain T3SS effectors from conserved effector locus (AvrE1, HopM1, HopAA1) and 13 other effectors (HopAK, HrpZ, HopJ, HrpA, HopAH2, HopC1, HopH1, HopZ3, HopAC1, HopI1, HopAZ1, HopBA1, and HopBF1). We detected nine coding sequences for T3SS effectors in the genome of strain P21 (HopAI1, HopAH1, HopAG1, AvrRpm1, HrpK, HopAW1, HopAU1, HopW, and HopAV1), which were absent in the genome of strain P16 (Fig. 4). Furthermore, we identified genes for three effectors in the genome of strain P21 (AvrRpm1, HopAW1, and HopAU1), which were not detected in any other P. syringae pv. *aptata* strain or known to be part of P. syringae pv. *aptata* effectorome thus far. The AvrRpm1 detected in strain P21 matches coding sequences of effectors secreted by P. syringae pv. *syringae* B728a strain with identity 99.54%. This effector was present only in genomes of strains from PG02, mainly in pv. *syringae*. On the other hand, the effector HopAU1 was matched with the same coding sequences of P. syringae pv. *theae* with an identity of 99.84% and was also detected in strain CFBP2116 from PG03. The HopAW1 was found only in seven other strains of PG02. The effector HopAW1 matches with multiple different P. syringae pathovars with more than 99% identity. The AvrRpm1 has a role in the phosphorylation of RIN4 protein and suppression of PAMP-triggered immunity and effector-triggered immunity (37). HopAW1 still has an unknown biochemical function. However, it belongs to the HopAS1 family (http://www.pseudomonas-syringae.org/hop_used_names.htm), which strongly impacts the virulence of P. syringae pv. *actinidae* and effector-triggered immunity in *Arabidopsis* (38). Interestingly, the HopAS1 effector was not present in any of the strains from PG02 (Fig. 4). HopAU1 in P. syringae pv. *phaseolicola* is characterized as an effector involved in the late stage of infection with the main role in modifying the apoplast into a replication-permissive niche, while *ΔhopAU1* strains couldn't reach wild-type growth level in plant apoplast (39). Additionally, the *ΔhopAU1* showed a constant level of expression during the early stages of infection and also altered expression levels corresponding to transcriptional regulators involved in response to environmental changes and induction of virulence factors such as phytotoxins and T3SEs (21). Future investigations of the genomes or secretomes of P. syringae pv. *aptata* strains should complement available data about the T3S effector repertoire obtained in draft genomes and their actual secretion in apoplast-like growth conditions.

We revealed 29 putative T3SS effectors for strain P21, among which 26 are already known and confirmed by BLASTp analyzes of their protein sequences listed in Fig. 4. Three putative proteins, which did not have BLAST hits with already known T3SS effectors for P. syringae complex, are pantothenate kinase, VapC endonuclease, and RAQPRD integrative conjugative protein. They could represent candidates for novel T3SS effector identification. For strain P16, predictive T3SS effector analysis showed 20 putative effectors, among which 16 effectors were confirmed by BLASTp analysis (Fig. 4). The other four proteins represent putative effectors, such as VapC endonuclease, phospholipase, pantothenate kinase, and pectate lyase. All coding sequences for putative novel effectors had high AUPRC scores in the range of 0.646 (VapC endonuclease) to 0.957 (Phospholipase). In addition, Effectidor analysis revealed approximately 30 putative novel effector-like proteins (AUPRC score above 0.5), suggesting that there is a much broader repertoire of effectors within the P. syringae complex than previously known.

All tested P. syringae strains, including P16 and P21 and other P. syringae pv. *aptata* strains have the *inaZ* gene for ice nucleation activity, the virulence sensor histidine kinase (*phoQ*), and virulence master regulator complexes (*algU/rpoE*, *csrA1A2*, *gacA*, and *bvgAS*) (Fig. 3). In addition, it should be noted that siderophores represent virulence factors important for the pathogen's survival in an iron-limited environment. Genes encoding the receptors for siderophores such as ferripyoverdin, ferripyochelin, ferrichrome, ferrienterobactin, ferric-anguinibactin, and ferric-pseudobactin BN8/BN7 were found in the genomes of all tested strains.

We did not detect any putative phytotoxins with the Prokka annotation. However, BLAST atlas analysis of P16 and P21 strains and reference strain B728a, a known producer of phytotoxins and closely related strains from the same phylogroup of the P. syringae complex, revealed the presence of the *syrD* gene encoding syringomycin in P16 and P21, with 98.24% and 97.06% identity with *syrD* in B728a, respectively. Genes for syringopeptin (*sypA*, *sypB* and *sypC*) were also detected in both Ptt strains and validated with BLAST hits against B728a with high identity (85% to 97%).

All analyzed 102 *P. syringe* genomes, including P. syringae pv. *aptata* strains possess sequences for the production of the plant hormone auxin (indole-3-acetic acid; IAA) (*iaaM*), and an enormous majority possess the gene for utilization of GABA (*gabP*) (Fig. 3). Auxin plays an important role in plants, affecting processes such as cell division, elongation, and fruit development, but overproduction and a high level of IAA can also promote disease susceptibility (40). Auxin-mediated sensitivity to disease caused by P. syringae pv. *tomato* DC 3000 was demonstrated to arise due to inhibition of host defenses (e.g., suppression of salicylic acid signaling network) (41). Draft genomes of the examined Ptt strains revealed coding sequences for GABA utilization (*gabP*) by GABA permease (Fig. 3). The plant defense mechanism of producing GABA, the most abundant amino acid in the apoplast, acts as a response to inhibiting T3SS expression (42). Utilization of GABA by GabP permease could directly attenuate the induction of plant defenses mechanisms in hosts where delivery of T3SSEs leads to host immunity responses triggered by effectors (43). Another important factor for plant disease development is motility-mediated virulence in P. syringae (44). Deletion of master regulators for swarming, swimming, or twitching motility of various P. syringae pathovars resulted in a reduction or a low level of the virulence potential (31, 45). We demonstrated that draft genomes of the examined Ptt strains contain various motility coding sequences, suggesting that strong motility-mediated colonization could be an important factor affecting the virulence of P. syringae pv. *aptata*. This is in accordance with results obtained from the swarming motility assay, where both strains (P16 and P21) performed positive swarming motility (8).

### Conclusions.

Analysis of draft genomes of two P. syringae pv. *aptata* strains in comparison with 100 other related Ptt genomes reveals key features of this pathovar required for dissemination in the phyllosphere of sugar beet. In our previous studies performed in greenhouse conditions, the P16 and P21 strains were established as distinct regarding their pathogenic potential. Evaluation of their draft genomes clearly demonstrates the high similarity of the repertoire of putative features required for disease occurrence. However, the T3SS effector analysis revealed notable differences in the repertoire of T3SS effectors between P16 and P21 strains, which can be responsible for such huge divergences obtained in greenhouse experiments and virulent phenotypes among strains. The T3SS effector analysis provides valuable data about confirmed effector repertoire as well as putative and novel T3SS effectors, suggesting the greater role of T3SS in P. syringae pv. *aptata* virulence and targeting the specific proteins which could be further investigated as candidates for novel effectors.

## MATERIALS AND METHODS

### Library preparation and genome sequencing.

Two strains of P. syringae pv. *aptata*, P16 and P21, were cultured on King's B medium (Titan Biotech Ltd.) and incubated at 27°C overnight, while DNA extraction for genome sequencing was performed using the Zymo BIOMICS Miniprep kit (Irvine, CA 92614, USA). Extracted DNA was sent to the CosmosID company (Rockville, MD, USA) for genome sequencing and annotation. Libraries were prepared using the Ion Xpress Plus Fragment Kit (Thermo Fisher Scientific) and quantified with Qubit (Thermo Fisher Scientific). Clonal amplification, purification, and library loading on an Ion 540 chip (Thermo Fisher Scientific) were performed using the Ion Chef System together with the Ion 540 Kit-Chef (Thermo Fisher Scientific). Sequencing with read lengths of 200 bp was carried on the Ion S5 XL System (Thermo Fisher Scientific). The genome sequences of P. syringae pv. *aptata* P16 and P21 were made publicly available at the NCBI GenBank (Acc. No. JAHCZG000000000.1 and JAHDTA000000000.1).

### Genome annotations.

Raw single-end reads were trimmed and processed using a BBDuk from BBtools v. 36.49 with a reading quality trimming parameter of 20 for isolates (46). The trimmed fastQ files were assembled using SPAdes v. 3.9.0 with the –careful parameter, while all other parameters were default (47). The resulting contigs were filtered for length using reformat from BBtools v. 36.49 and only contigs that were at least 500 bp were retained. The genome size of P16 and P21 was calculated by counting k-mer frequency of the raw read data and compared with the raw read data of other P. syringae strains available from NCBI. The Jellyfish v. 2.2.6 tool was used for counting of k-mers (48). CheckM's v. 1.0.13 was used with default parameters to generate a genome bin plot (49). The genome-wide annotation was performed with Prokka, which uses the Prodigal tool (50) to identify the coordinates of candidate genes and for the prediction of coding sequences, compares them with large databases with known sequences: NCBI+ blastp, UniProt, RefSeq and a series of hidden Markov model (HMM) profile databases, including Pfam and TIGRFAMs (51). The HMM is performed using hmmscan from the HMMER 3.1 package (52). The CDS were classified into different groups based on their roles in the cell, with reference to orthologous groups (COGs; http://www.ncbi.nlm.nih.gov/COG/). Lastly, for the identification of antimicrobial resistance (AMR) genes and virulence factors (VFs), the assembled genomes were screened against the Resfinder (53), AMR and VFDB (54). AMR and VF genes were considered present if their sequences matched with the assembled genome at >90% nucleotide identity and >60% alignment coverage of the gene's sequence length.

### Comparative genomics.

The genome sequences were compared to other genomes of strains/pathovars from the P. syringae species complex (Table 1). The used strains belong mostly to the PG02, closely related to P. syringae pv. *aptata* and consisting of ubiquitous and virulent strains with a great potential to cause plant disease epidemics. Besides strains from PG02, the representative strains of PGs 01, 03, 04, 07, 09, 10, and 13 were also included in the comparative analysis. For the ANI analysis, statistics were first calculated for all 102 genomes using the assembly-stats (https://github.com/sanger-pathogens/assembly-stats) package, and three genomes (strain code: NZIPFR-VIR1, WSPS007, and DSM50252) were excluded that had a value of N50 < 10 kbp, which was the necessary minimum for FastANI analysis. The genome comparisons were made between all pairwise combinations of strains using FastANI (22), and all strain pairs were tested using the “many to many” method in FastANI and by using the “–matrix” option. Earlier studies have shown that an ANI value of 94% to 96% corresponds to the recommended DNA-DNA hybridization species cutoff of 70% (55). ANIclustermap v1.2.0 (https://github.com/moshi4/ANIclustermap) was implemented to visualize the results.

Pangenome analysis was performed using Roary v3.13.0 (56) with previously annotated selected 102 genomes with Prokka annotation as input files in GFF format. We used all default parameters (56), except the parameters group limit which is increased to 60,000 clusters. The results were visualized using the script roary_plots.py. and create_pan_genome_plots.R software of the Roary package. The genes were classified as the core if found in >99% of genomes, while genes present in 95% to 99%, 15% to 95%, and less than 15% were considered soft-core, shell, and cloud genes, respectively. The approximately maximum likelihood phylogenetic tree was built using the core_genome_alignment.aln file in FastTree 2.1 software (57). The pan-genome was constructed from inputted genome assemblies and then determined the gene set present in each genome assembly. That gene set (output file: gene_presence_absence) was used for reporting results about gene differences involved in pathogenic features among P16 and P21 strains and all other P. syringae strains. Genes encoding T3SSEs were excluded from this analysis and were subjected to independent analysis. Phyton (v. 3.8.10) was used for image generation in the Jupyter Notebook tool. BLAST atlases were generated by Gview server (https://server.gview.ca, [58]) by carrying out genome-wide blastn searches in order to compare P16 and P21 to the genome of reference strain B728a (GenBank accession no. NC_007005). Homologous genes on each genome reporting a BLAST hit above the threshold cut-off (80% identity, minimum HSP length of 100 bp, and expected value of 1e^−10^) were considered a valid match.

### Type III secretion effectors identification.

The type III secretion system effectors for all 102 genomes are predicted and identified using the Effectidor web server (https://effectidor.tau.ac.il/) prediction pipeline, which represents a new machine-learning-based prediction tool (59). The input file represented genome annotations, and the effector input file consisted of an annotated T3SS effector from genome annotations, which trained the pipeline for T3SS effector detection. A total of 51 obligatory features were extracted from a mandatory input file, including all the bacterium DNA ORF sequences in a FASTA format. These features include the GC content, protein length, relative frequencies of amino acids in the full protein and the N-terminal region, homology to known T3Es in other bacteria and the analyzed strain, etc. The area under the precision-recall curve (AUPRC) was used as a scoring method, while results were obtained using 0.5 as a threshold for identifying effectors. AUPRC value is sensitivity and specificity value that machine learning software utilizes to accurately classify components of a set into two precisely divided categories and indicates if the component represents T3S effector or not. The AUPRC score reflects inference precision averaging over all possible cutoffs. The AUPRC of Effectidor on these data were 1.0. Predicted effectors were subsequently blasted using NCBI BLASTp tool (https://blast.ncbi.nlm.nih.gov/Blast.cgi) to validate their detection.
